# AMPK Activation as a Protective Mechanism to Restrain Oxidative Stress in the Insulin-Resistant State in Skeletal Muscle of Rat Model of PCOS Subjected to Postnatal Overfeeding

**DOI:** 10.3390/biomedicines11061586

**Published:** 2023-05-30

**Authors:** Bojana Mićić, Ana Djordjevic, Nataša Veličković, Sanja Kovačević, Teodora Martić, Djuro Macut, Danijela Vojnović Milutinović

**Affiliations:** 1 Department of Biochemistry, Institute for Biological Research “Siniša Stanković”-National Institute of the Republic of Serbia, University of Belgrade, 142 Despot Stefan Blvd, 11060 Belgrade, Serbia; bojana.micic@ibiss.bg.ac.rs (B.M.); djordjevica@ibiss.bg.ac.rs (A.D.); nvelickovic@ibiss.bg.ac.rs (N.V.); sanja.kovacevic@ibiss.bg.ac.rs (S.K.); ttedorix@gmail.com (T.M.); 2 Clinic for Endocrinology, Diabetes and Metabolic Diseases University Clinical Centre of Serbia, Faculty of Medicine, University of Belgrade, Doktora Subotića 13, 11000 Belgrade, Serbia; djmacut@gmail.com

**Keywords:** PCOS, postnatal overfeeding, 5α-dihydrotestosterone, obesity, insulin resistance, skeletal muscle, AMPK, lipid metabolism, oxidative stress, mitochondrial β-oxidation

## Abstract

Polycystic ovary syndrome (PCOS) is a common endocrinopathy in women of reproductive age, often associated with obesity and insulin resistance. Childhood obesity is an important predisposing factor for the development of PCOS later in life. Being particularly interested in the interplay between prepubertal obesity and hyperandrogenemia, we investigated the effects of early postnatal overfeeding, accomplished by reducing litter size during the period of suckling, on energy sensing and insulin signaling pathways in the gastrocnemius muscle of a rat model of PCOS-induced by 5α-dihydrotestosterone (DHT). The combination of overfeeding and DHT treatment caused hyperinsulinemia and decreased systemic insulin sensitivity. Early postnatal overfeeding induced defects at critical nodes of the insulin signaling pathway in skeletal muscle, which was associated with reduced glucose uptake in the presence of hyperandrogenemia. In this setting, under a combination of overfeeding and DHT treatment, skeletal muscle switched to mitochondrial β-oxidation of fatty acids, resulting in oxidative stress and inflammation that stimulated AMP-activated protein kinase (AMPK) activity and its downstream targets involved in mitochondrial biogenesis and antioxidant protection. Overall, a combination of overfeeding and hyperandrogenemia resulted in a prooxidative and insulin-resistant state in skeletal muscle. This was accompanied by the activation of AMPK, which could represent a potential therapeutic target in insulin-resistant PCOS patients.

## 1. Introduction

Polycystic ovary syndrome (PCOS) is the most common endocrine disorder in women of reproductive age. Despite its high prevalence of 4–20% and numerous reproductive, metabolic, and psychological implications, the syndrome remains poorly understood among clinicians, in part because of its complexity and unclear underlying pathophysiologic mechanisms [1,2]. Based on its diagnostic criteria, which include chronic anovulation, hyperandrogenism, and polycystic ovarian morphology [3], the syndrome is generally considered a primarily reproductive disorder linked with reduced fertility potential, increased pregnancy complications, and endometrial dysfunction [4,5]. Besides this, it is well known that PCOS is associated with adverse metabolic conditions such as obesity, insulin resistance, and dyslipidemia, and PCOS patients have a higher risk of the development of type 2 diabetes mellitus (T2DM) and gestational diabetes [3,6].

The initial manifestations of PCOS occur in prepuberty, and numerous studies indicate that during this period, obesity stimulates the progression of the syndrome in puberty and adolescence [7,8]. Modern lifestyle, reflected in poor dietary habits and decreased physical activity, is usually considered the main cause of the current obesity epidemic [9]. However, nutritional status in early childhood is known to play an important role in the development of overweight, obesity, and associated chronic diseases [9,10]. Early postnatal overfeeding leads to the misprogramming of the hypothalamic-pituitary-adrenal (HPA) and hypothalamic-pituitary-gonadal (HPG) axes, resulting in a lifelong increase in food consumption, susceptibility to decreased glucose and insulin tolerance, shortened onset of puberty and reproductive underperformance throughout lifetime [9]. In rodents, postnatal overfeeding can be easily achieved by reducing litter size, making rodents raised in a small litter a useful model for studying the consequences of early-life overnutrition [9,11,12,13].

The pathophysiological mechanisms of PCOS are complex and not fully understood. Many factors, including genetic susceptibility, maternal milieu, and postnatal environment, likely synergize in the development and expression of PCOS [6]. Hyperandrogenism is considered to play an essential role in the development of most reproductive and metabolic perturbations associated with PCOS. It was shown that prenatal or early postnatal exposure to androgen excess predisposes the development of a PCOS-like phenotype in adulthood [14]. Consistent with this, there are literature data suggesting that female fetuses exposed to high levels of androgens during the intrauterine period develop clinical features of PCOS later in life [15]. Since it is not possible to perform controlled studies in humans to observe the fetal consequences of high maternal androgens, different animal models of prenatal androgen treatment have been developed [16]. These animal studies suggest that maternal hyperandrogenism at a critical stage of fetal development may cause permanent changes in fetal physiology that can trigger the development of PCOS later in adulthood. In addition, treatment with different androgens in postnatal period in rodents also results in the adult onset of PCOS-like traits [16]. Overweight and obesity, with a prevalence of up to 80% among women with PCOS, is one of the key factors contributing to the worsening of not only metabolic disturbances, but also reproductive ones [17]. Moreover, it was shown that increased adiposity during the prepubertal period aggravates hyperinsulinemia and consequently stimulates androgen production and increases androgen levels. A new hypothesis supports the concept that a “PCOS secondary to obesity” may exist, and that prepubertal obesity might be a preconditioned state for hyperandrogenemia [18].

The imbalance of sex hormones, especially androgens, has a strong influence on the development of T2DM in both men and women. In women, the risk of developing T2DM increases with the exacerbation of hyperandrogenism, which is particularly evident in hyperandrogenic disorders such as PCOS. Women with PCOS are two to eight times more likely to develop metabolic disorders such as glucose intolerance, insulin resistance, metabolic inflammation, and T2DM compared with BMI-matched controls [19,20].

Although the etiology of PCOS remains elusive, there is strong evidence that in addition to elevated androgen levels, insulin resistance plays an important role in it [21,22], with a prevalence of up to 95% in PCOS patients [23]. Insulin resistance of peripheral metabolically active tissues, adipose tissue and skeletal muscle, contributes to PCOS to different extents, with likely similar defects in metabolic pathways and different time courses [2].

Since skeletal muscles account for approximately 40% of total body mass and are capable of disposing of up to 80% of blood glucose, they are considered a key factor in driving the adverse metabolic phenotype in PCOS [2,24,25]. It is known that insulin resistance in skeletal muscle can occur years before the onset of systemic hyperglycemia [26]. In addition to carbohydrates, skeletal muscles are also capable of using lipids as a fuel source. The ability of a muscle to switch between the oxidation of lipids to oxidation of carbohydrates is known as metabolic flexibility. A significant decrease in metabolic flexibility is associated with both obesity and PCOS [25]. Metabolic inflexibility implies the inability of a muscle to adequately activate glucose metabolism in response to a glucose load. However, defects in energy-sensing pathways, defects in mitochondrial fuel selection, intracellular or extracellular lipid accumulation leading to lipotoxicity, and oxidative stress are also factors that have been shown to impair metabolic flexibility and contribute to the development of insulin resistance in skeletal muscle [2,25].

Understanding the molecular mechanisms of insulin resistance in PCOS, particularly those responsible for the synergy between obesity and insulin resistance in deficiency of skeletal muscle function, is essential for prompt diagnosis and improvement of available and development of new therapeutic approaches.

Although PCOS is a human disorder, different animal models are being developed that are essential for mechanistic research that forms the basis for both understanding the etiology of the disease and improving the treatment of patients.

With the aim to better understand the molecular basis of the interplay between obesity and insulin resistance in skeletal muscle in PCOS, we investigated the expression of key components of the insulin signaling pathway, main energy sensor—AMP-activated kinase (AMPK), enzymes involved in lipid mobilization and β-oxidation, as well as markers of oxidative damage and inflammation in the gastrocnemius muscle of an established Wistar rat 5α-dihydrotestosterone (DHT)-induced model of PCOS, additionally challenged by postnatal overfeeding gained through litter size reduction.

## 2. Materials and Methods

### 2.1. Study Design and Animals

This study was performed on Wistar rats. At the beginning of the experiment, females were 2 days old, and litter size was manipulated to induce early postnatal overfeeding (10 or 3 pups with lactating mothers, representing normal and small litters, respectively). At weaning, placebo pellets without active substance or pellets containing 7.5 mg 5α-DHT (Innovative Research of America, Sarasota, FL, USA) were implanted in the neck region of the animals. In this way, four experimental groups were formed: normal litter-raised animals with placebo pellets (NL-placebo, *n* = 6 animals), normal litter-raised animals with DHT pellets (NL-DHT, *n* = 6 animals), small litter-raised animals with placebo pellets (SL-placebo, *n* = 6 animals), and small litter-raised animals with DHT pellets (SL-DHT, *n* = 6 animals). The DHT-containing pellets continuously released 83 μg of DHT daily for 90 days, a dose consistent with androgen levels observed in PCOS patients [27,28].

After weaning and after pellets were implanted, the animals were housed three per cage, under standard conditions and had *ad libitum* access to standard laboratory chow and tap water.

At the end of the 90-day treatment, animals were killed after six hours fast in the diestrus phase. The decapitation was performed using a guillotine (Harvard Apparatus, Holliston, MA, USA).

The Ethics Committee for the Use of Laboratory Animals of the Institute for Biological Research “Siniša Stanković” has approved the study under permit No. 01-02/19. All experimental procedures complied with the EEC Directive 2010/63/EU on the protection of animals used for experimental and other scientific purposes.

### 2.2. Assessment of Systemic Insulin Sensitivity

At decapitation, blood from the trunk was collected at room temperature and incubated for coagulation (40 min). The concentration of glucose was determined using the Accu-Chek apparatus (Roche, Mannheim, Germany). Blood serum was obtained by centrifugation (1600× *g*, 10 min, 14 °C, Eppendorf 5804/R, Hamburg, Germany) and stored at −70 °C for further analysis. Serum insulin concentration was measured commercially at the Institute for Application of Nuclear Energy (INEP), Belgrade, Serbia, using an in-house developed radioimmunoassay (RIA) kit, with a detection limit of 0.06 mU/L, while intra- and inter-assay coefficients of the variation were 2.5% and 7.7%, respectively. According to the information provided by INEP, the sensitivity (detection limit) of the RIA insulin test was determined as twice the standard deviation value for the “0” standard, while the intra-assay and inter-assay coefficients of variation of the sera were calculated for three different insulin concentrations. Serum insulin concentration and glycemia at the time of decapitation were used to calculate the Quantitative insulin sensitivity check index (QUICKI), according to the following formula: 1/[log insulin (μU/mL) + log glucose (mg/dL)].

An intraperitoneal glucose tolerance test (ipGTT) was performed at the end of the study. After a 6-h fast, animals were intraperitoneally administered without anesthesia with 2 g glucose per kg body weight. Samples of blood were collected from the tip of the tail at different time points after glucose injection (0, 15, 30, 60, 90, 120 min). Glucose concentration used to plot glucose curves was determined using the Accu-Chek apparatus.

### 2.3. Preparation of Whole Cell Extracts and Western Blot Analyses

After decapitation, gastrocnemius muscle was excised, washed in 0.9% saline, dried, and stored at −70 °C until use. Muscle tissue was homogenized in 4 volumes of ice-cold RIPA buffer (50 mM Tris-HCl, pH 7.4, containing 150 mM NaCl, 10 mM EDTA-Na_2_, 0.5% Triton X, 1% NP40, 0.1% SDS, 2 mM dithiothreitol (DTT) and protease and phosphatase inhibitors), using the Janke-Kunkel Ultra Turrax T25 (IKA^®^, Staufen, Germany). Homogenates were sonicated for 3 × 5 s, 1 A, 50/60 Hz (Hielscher Ultrasound Processor, Teltow, Germany), incubated on ice for 30 min with frequent agitation and vortexing, and centrifuged (16,000× *g*, 35 min, 4 °C). Supernatants were used as whole cell extracts.

The obtained whole cell extracts were mixed 1:1 with 2 × Laemmli buffer and boiled for 5 min after the determination of protein concentration by the Lowry method [29].

Boiled protein samples were resolved on 8–12% sodium dodecyl sulfate-polyacrylamide gels (SDS-PAGE) and transferred to polyvinylidene difluoride (PVDF) membranes (Immobilon-FL, Millipore, Billerica, MA, USA) in the Trans-Blot Turbo Transfer System (Bio-Rad Laboratories, Hercules, CA, USA). Membranes were blocked with 5% bovine serum albumin or nonfat milk and then probed with primary antibodies against the following proteins: insulin receptor substrate 1 phosphorylated at Ser 307 (pIRS1-Ser307; 1:500, ab5599), peroxisome proliferator-activated receptor-gamma coactivator 1α (PGC1α; 1:1000, ab54481), superoxide dismutase (SOD2; 1:2000, ab13533), all purchased from Abcam, Cambridge, UK; pAkt1/2/3-Ser473 (1:500, sc-514032), pAkt1/2/3-Thr308 (1:500, sc-271966), Akt1/2/3 (1:500, sc-81434), AMP-activated kinase (AMPKα1/2; 1:1000, sc-25792), sirtuin 1 (SIRT1; 1:500, sc-15404), all purchased from Santa Cruz Biotechnology, Dallas, TX, USA; GLUT4 (1:500, 2213s), pAMPKα-Thr172 (1:1000, 2535s), all purchased from Cell Signaling, Danvers, MA, USA, and Perilipin-2 (1:1000, NB110-40877) purchased from Novus Biologicals, Centennial, CO, USA). To correct protein loading, membranes were probed with anti-β-tubulin antibody. Anti-β-tubulin antibody was a WA3 mouse monoclonal antibody raised against bovine brain β-tubulin (Dr. Ursula Euteneuer). Prior to incubation for 90 min with proper mouse (1:30,000, ab97046) or rabbit (1:20,000, ab6721) secondary antibodies conjugated with HRP (Abcam, Cambridge, UK), membranes were washed in PBS containing 0.1% Tween-20. Enhanced chemiluminescence method was used to visualize and quantify immunoreactive bands by iBright FL1500 Imaging System and Analysis Software (Thermo Fisher Scientific, Waltham, MA, USA).

### 2.4. Isolation of RNA and RT-qPCR

Total RNA was isolated from gastrocnemius muscle following the manufacturer’s instructions, using TRIzol Reagent (Invitrogen, Waltham, MA, USA). The concentration of extracted RNA was assessed at 260 nm by spectrophotometry (Nano Photometer N60, Implen GmbH, Munich, Germany).

Total RNA (2 µg) was transcribed to complementary DNA (cDNA) by the High Capacity Reverse Transcription Kit (Applied Biosystems, Foster City, CA, USA) according to the instructions provided by the manufacturer. Obtained cDNAs were kept at −70 °C until use.

All real-time PCR reactions were performed on the QuantStudio™ Real-Time PCR System (Applied Biosystems, Foster City, CA, USA) in a total volume of 10 µL. SYBR Green reaction mixes consisted of SsoAdvanced Universal SYBR Green Supermix (Bio-Rad Laboratories, Hercules, CA, USA), specific primer pairs, and 20 ng of cDNA template, while TaqMan reaction mixes included TaqMan^®^ master mix, and gene expression assay, together with cDNA. Conditions of the reactions were incubation (2 min, 50 °C), 10 min at 95 °C, 40 cycles at 95 °C for 15 s, and 60 °C for 60 s. To validate the synthesis of a single PCR product for the SYBR Green reactions, we analyzed melting curves obtained at the end of each experiment. For each target gene, no template controls were used. The comparative 2^−∆∆Ct^ method was used to calculate relative gene expression.

Adipose tissue triglyceride lipase (ATGL) expression was determined with TaqMan gene expression FAM labeled probe (*Pnpla2*, Rn01479969_m1), with TATA box binding protein (*Tbp*, Rn01455646_m1) as a reference gene. The expression of Stearoyl CoA Desaturase 1 (*Scd1*, F: 5′-TGG TGC TCT TTC CCT GTT TGC-3′; R: 5′-TGG GCT TTG GAA GGT GGA CA-3′), Carnitine palmitoyltransferase 1b (*Cpt1b*, F: 5′-CCA GGC AAA GAG ACA GAC TTG-3′; R: 5′-GCC AAA CCT TGA AGA AGC GA-3′), Fatty acid transport protein 1 (*Fatp1* F: 5′-CCC AAG TGG ATA CAA CAG GCA-3′, R: 5′-GGT CTA GAA AGA AGA GCC GGT C-3′), Interleukin 1β (*Il1b*, F: 5′-AGC AGC TTT CGA CAG TGA GG-3′; R: 5′-CTC CAC GGG CAA GAC ATA GG-3′), and Interleukin 6 (*Il6*, F: 5′- GTT TCT CTC CGC AAG AGA CTT-3′; R: 5′-ATA CTG GTC TGT TGT GGG TGG-3′) was analyzed by SYBR^®^ Green qPCR, with all primers purchased from Microsynth, Balgach, Switzerland. Hypoxanthine guanine phosphoribosyl transferase was used as a reference gene (*Hprt*, F: 5′-CAG TCC CAG CGT GAT TA-3′; R: 5′-AGC AAG TCT TTC AGT CCT GTC-3′) (Invitrogen, Waltham, MA, USA).

### 2.5. Measurement of Lipid Peroxidation

Lipid peroxidation in muscle tissue was assessed using a Lipid Peroxidation (MDA) Assay Kit (Merck, Darmstadt, Germany), according to the manufacturer’s instructions. Briefly, 10 mg of tissue was homogenized on ice in MDA Lysis Buffer containing BHT and centrifuged. The supernatant was mixed with TBA solution and incubated at 95 °C for 60 min. Absorbance was measured at 532 nm, and the concentration of malondialdehyde in samples was extrapolated from its standard curve.

### 2.6. Statistical Analyses

All data are presented as mean ± standard error of the mean (SEM). The effects of factors (litter size and DHT) and the interaction between them were determined by a two-way ANOVA. In the case when a significant interaction between factors was detected, the Tukey *post hoc* test was applied to assess inter-group differences. Statistical significance was accepted at *p* < 0.05. The analyses were conducted with GraphPad Prism 8 (San Diego, CA, USA) and STATISTICA 7.0 (StatSoft Inc., Tulsa, OK, USA) software packages.

## 3. Results

### 3.1. Parameters of Systemic Insulin Sensitivity

**Figure 1:**
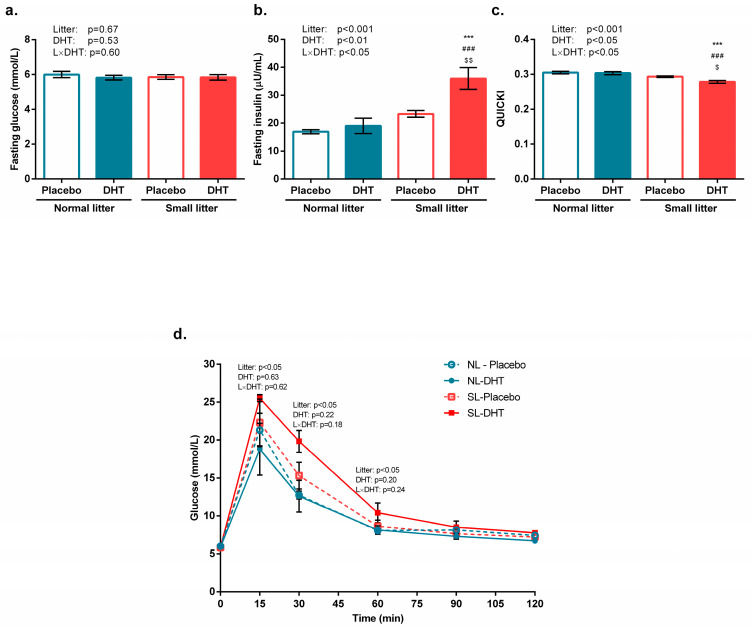
Glycemia and systemic insulin sensitivity parameters of placebo and 5α-dihydrotestosterone (DHT)-treated rats raised in small and normal litters (SL and NL, respectively). (**a**) Blood glucose levels at decapitation; (**b**) Serum insulin levels at decapitation; (**c**) Quantitative insulin-sensitivity check index (QUICKI) calculated from fasting insulin and glucose levels; (**d**) Glucose curve from ipGTT performed before the end of the experiment. Data are provided as mean ± SEM (*n* = 6 animals per group). To determine the effects of litter size reduction and DHT treatment, as well as their interaction, a two-way ANOVA was performed, followed by the Tukey *post hoc* test in a case of interaction between the factors. Statistical significance was accepted at *p* < 0.05. Symbols represent a significant difference in comparison with NL-Placebo group, NL-DHT, or SL-Placebo group (*** *p* < 0.001; ^###^ *p* < 0.001; ^$$^ *p* < 0.01; ^$^ *p* < 0.05, respectively).

Litter size reduction and DHT treatment did not affect fasting glucose levels in any of the analyzed groups, as shown in Figure 1a.

However, a significant effect of litter size (F(1, 20) = 22.13, *p* < 0.001), DHT (F(1, 20) = 8.86, *p* < 0.01), and their interaction(F(1, 20) = 4.58, *p* < 0.05) on fasting insulin concentration were detected. As presented in Figure 1b, the Tukey *post hoc* test showed increased insulin concentration in SL-DHT compared to NL-Placebo, NL-DHT, and SL-Placebo group (*** *p* < 0.001, ^###^ *p* < 0.001, and ^$$^ *p* < 0.01, respectively).

Systemic insulin sensitivity was assessed using QUICKI. A significant effect of litter size (F(1, 20) = 27.05, *p* < 0.001), DHT treatment (F(1, 20) = 5.50, *p* < 0.05), and their interaction (F(1, 20) = 6.46, *p* < 0.05) was observed. As revealed by the Tukey *post hoc* test and shown in Figure 1c, the QUICKI index was increased in the SL-DHT group in comparison with NL-Placebo, NL-DHT, and SL-Placebo (*** *p* < 0.001, ^###^ *p* < 0.001, and ^$^ *p* < 0.05, respectively).

A main effect of litter size reduction on increased glucose excursion in the SL-DHT group was detected at different time points, as shown in Figure 1d (15 min: F(1, 17) = 4.57, *p* < 0.05; 30 min: F(1, 17) = 8.02, *p* < 0.05; 60 min: F(1, 17) = 4.07, *p* < 0.05).

### 3.2. Insulin Signaling and Glucose Uptake

**Figure 2:**
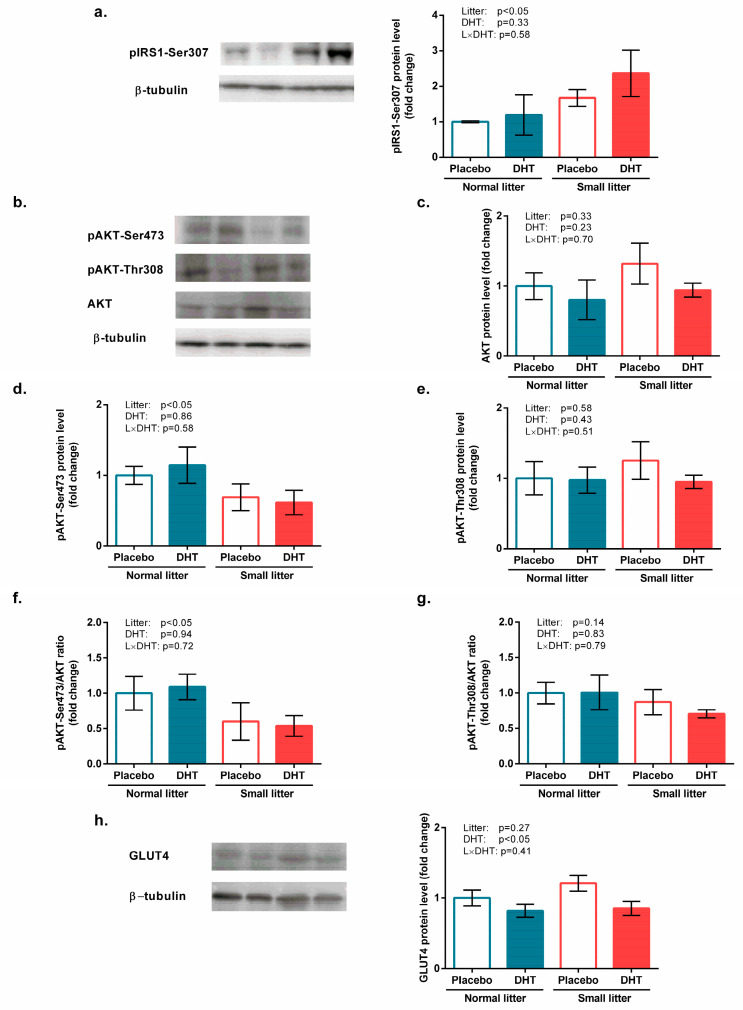
Insulin signaling and glucose uptake in the gastrocnemius muscle of rats implanted with placebo or DHT pellets and raised in SL or NL. (**a**) pIRS1-Ser307 protein levels with a representative Western blot; (**b**) Representative Western blots for pAKT-Ser473, pAKT-Ser308, and total AKT; (**c**) Protein level of total AKT; (**d**) Protein level of pAKT-Ser473; (**e**) Protein level of pAKT-Thr308; (**f**) Relative ratio of pAKT-Ser473/AKT; (**g**) Relative ratio of pAKT-Thr308/AKT; (**h**) Protein level of GLUT4 with a representative Western blot. Protein levels were determined in whole-cell extract of the gastrocnemius muscle and standardized to β-tubulin. Data from 6 animals/group are provided as mean ± SEM. A two-way ANOVA was used to assess the effects of DHT and litter size and their interaction. Statistical significance was accepted at *p* < 0.05.

Effects of applied treatments on the insulin signaling pathway in the gastrocnemius muscle were observed at their onset. Namely, a significant effect of litter size (F(1, 20) = 4.18, *p* < 0.05, Figure 2a) was observed for IRS1 phosphorylation on Serine 307. The effect of litter size was also observed for activating phosphorylation of AKT at Serine 473 (F(1, 20) = 4.77, *p* < 0.05; Figure 2d) and pAKT-Ser473/AKT ratio (F(1, 20) = 5.04, *p* < 0.05; Figure 2f). The protein content of total AKT (Figure 2c) and pAKT with activating phosphorylation at Threonine 308 (Figure 2e), as well as the pAKT-Thr308/AKT ratio, remained unchanged after the treatments (Figure 2g).

A significant effect of DHT treatment on the protein level of GLUT4 in the gastrocnemius muscle (F(1, 20) = 6.51, *p* < 0.05; Figure 2h) was also revealed.

### 3.3. Energy Sensing and Lipid Metabolism

**Figure 3:**
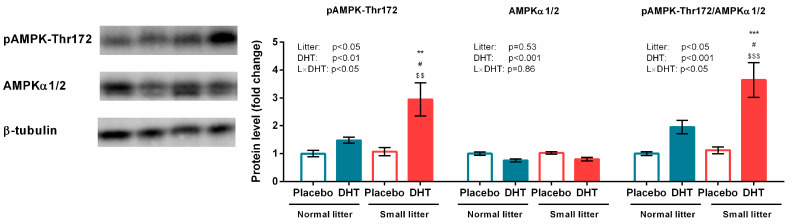
Phosphorylated and total AMPK protein levels, and their relative ratio, in the gastrocnemius muscle of rats implanted with placebo or DHT pellets and raised in SL or NL. Protein levels were determined in whole-cell extract of the gastrocnemius muscle and standardized to β-tubulin. Representative Western blots are provided. Data from 6 animals/group are provided as mean ± SEM. A two-way ANOVA was used to assess the effects of DHT and litter size and their interaction, followed by the Tukey *post hoc* test in a case of interaction between the factors. Statistical significance was accepted at *p* < 0.05. Symbols represent a significant difference in comparison with NL-Placebo, NL-DHT, or SL-Placebo group (*** *p* < 0.001; ** *p* < 0.01; ^#^ *p* < 0.05; ^$$$^ *p* < 0.001; ^$$^ *p* < 0.01, respectively).

A significant effect of litter size (F(1, 20) = 5.87 *p* < 0.05), DHT treatment (F(1, 20) = 13.72, *p* < 0.01), and their interaction (F(1, 20) = 4.83, *p* < 0.05) was observed on AMPK activating phosphorylation at Threonine 172. Total protein expression of AMPK was affected only by DHT treatment (F(1, 20) = 17.04, *p* < 0.001). The relative ratio of phosphorylated and total AMPK forms was also affected by litter size (F(1, 20) = 7.02, *p* < 0.05), DHT treatment (F(1, 20) = 25.93, *p* < 0.001), and their interaction (F(1, 20) = 5.28, *p* < 0.05). As presented in Figure 3, the pAMPK-Thr172 protein level and the relative pAMPK-Thr172/AMPKα1/2 ratio were increased in the SL-DHT group compared with all other groups (** *p* < 0.01, *** *p* < 0.001 vs. NL-Placebo; ^#^ *p* < 0.05 vs. NL-DHT; ^$$^ *p* < 0.01; ^$$$^ *p* < 0.001 vs. SL-Placebo).

**Figure 4:**
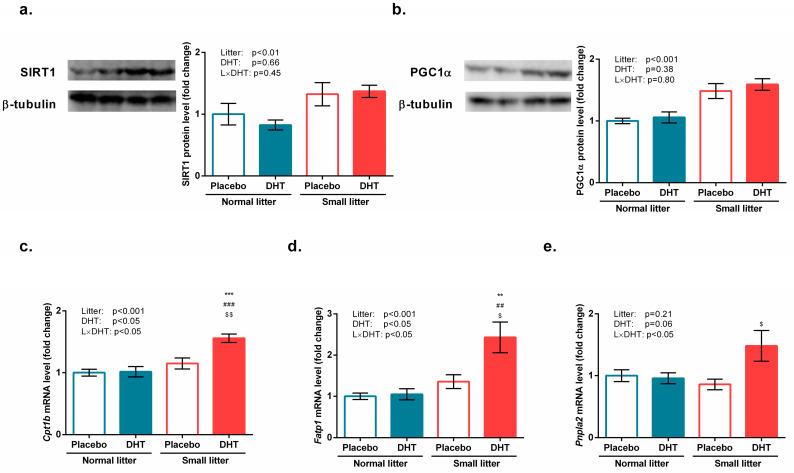
β-oxidation and fatty acid import controlling factors in the gastrocnemius muscle of rats implanted with placebo or DHT pellets and raised in SL or NL. Protein levels of (**a**) SIRT1 and (**b**) PGC1α with representative Western blots; mRNA levels of (**c**) *Cpt1*; (**d**) *Fatp1;* and (**e**) *Pnpla2*. Protein levels were determined in whole-cell extract of the gastrocnemius muscle and standardized to β-tubulin. Representative Western blots are provided. Data from 6 animals/group are provided as mean ± SEM. A two-way ANOVA was used to assess the effects of DHT and litter size and their interaction, followed by the Tukey *post hoc* test in a case of interaction between the factors. Statistical significance was accepted at *p* < 0.05. Symbols represent a significant difference in comparison with NL-Placebo group, NL-DHT group, or SL-Placebo group (*** *p* < 0.001; ** *p* < 0.01; ^###^ *p* < 0.001; ^##^ *p* < 0.01; ^$$^ *p* < 0.01; ^$^ *p* < 0.05, respectively).

A significant effect of litter size reduction on protein expression of SIRT1 (F(1, 20) = 9.18, *p* < 0.01) and PGC1α (F(1, 20) = 30.9, *p* < 0.001) has been detected, as presented in Figure 4a,b.

Litter size (F(1, 20) = 20.67, *p* < 0.001) and DHT treatment (F(1, 20) = 7.77, *p* < 0.05), as well as their interaction (F(1, 20) = 6.66, *p* < 0.05), affected the expression of the *Cpt1. Fatp1* gene expression was also affected by litter size (F(1, 20) = 15.74, *p* < 0.001), DHT treatment (F(1, 20) = 6.56, *p* < 0.05), and their interaction (F(1, 20) = 5.49, *p* < 0.05) in the gastrocnemius muscle of the treated animals. As revealed by the Tukey post hoc test and shown in Figure 4c,d, the mRNA expression of *Cpt1* and *Fatp1* was significantly elevated in the SL-DHT group as compared with all other groups (*** *p* < 0.001, ** *p* < 0.01 vs. NL-Placebo, ^###^ *p* < 0.001, ^##^ *p* < 0.01 vs. NL-DHT; ^$$^ *p* < 0.01, ^$^ *p* < 0.05 vs. SL-Placebo).

The expression of the ATGL encoding gene (*Pnpla2*) was affected by the interaction of DHT and litter size factors (F(1, 20) = 5.12, *p* < 0.05) and elevated in the SL-DHT group, as compared with the SL-Placebo group (^$^*p* < 0.05, Figure 4e).

**Figure 5:**
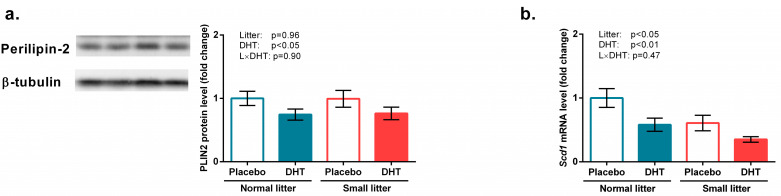
Factors involved in intramuscular triglycerides accumulation in the gastrocnemius muscle of rats implanted with placebo or DHT pellets and raised in SL or NL. (**a**) Perilipin-2 protein level with a representative Western blot; (**b**) mRNA levels of *Scd1.* Protein levels were determined in whole-cell extract of the gastrocnemius muscle and standardized to β-tubulin. Data from 6 animals/group are provided as mean ± SEM. A two-way ANOVA was used to assess the effects of DHT and litter size and their interaction. Statistical significance was accepted at *p* < 0.05.

As shown in Figure 5a, protein level of perilipin-2 was affected by DHT treatment (F(1, 20) = 4.87, *p* < 0.05), whereas litter size (F(1, 20) = 7.92, *p* < 0.05) and DHT (F(1, 20) = 9.32, *p* < 0.01) affected *Scd1* gene expression (Figure 5b).

### 3.4. Markers of Oxidative Stress and Inflammation

**Figure 6:**
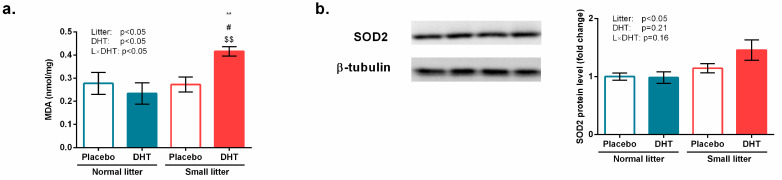
Markers of oxidative stress in the gastrocnemius muscle of rats implanted with placebo or DHT pellets and raised in SL or NL. (**a**) The amount of MDA in gastrocnemius tissue homogenate; (**b**) Protein levels of SOD2 with a representative Western blot. Protein levels were determined in whole-cell extract of the gastrocnemius muscle and standardized to β-tubulin. Data from 6 animals/group are provided as mean ± SEM. A two-way ANOVA was used to assess the effects of DHT and litter size and their interaction, followed by the Tukey *post hoc* test in a case of interaction between the factors. Statistical significance was accepted at *p* < 0.05. Symbols represent a significant difference in comparison with NL-Placebo, NL-DHT, and SL-Placebo groups (** *p* < 0.01; ^#^ *p* < 0.05; ^$$^ *p* < 0.01, respectively).

A significant effect on the amount of MDA in the gastrocnemius muscle, which represents a direct measure of lipid peroxidation, was detected for litter size (F (1, 20) = 5.69, *p* < 0.05), DHT treatment (F(1, 20) = 5.74, *p* < 0.05), as well as their interaction (F(1, 20) = 5.74, *p* < 0.05). Tukey *post hoc* test revealed a significant increase in MDA amount in SL-DHT, compared with NL-Placebo, NL-DHT, and SL-Placebo group (** *p* < 0.01; ^#^ *p* < 0.05; ^$$^ *p* < 0.01), as shown in Figure 6a. The protein level of SOD2 was affected by litter size reduction (F(1, 20) = 7.48, *p* < 0.05), as shown in Figure 6b.

**Figure 7:**
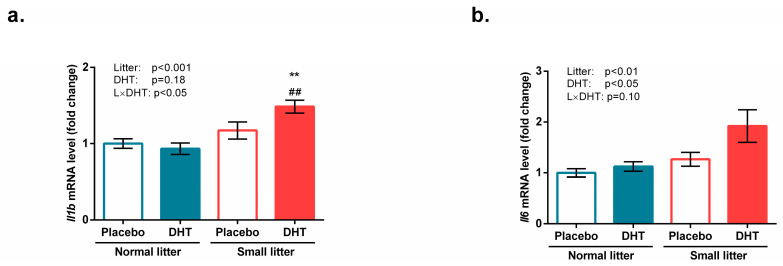
Markers of inflammation in the gastrocnemius muscle of rats implanted with placebo or DHT pellets and raised in SL or NL. mRNA levels of (**a**) *Il1b* and (**b**) *Il6.* Data from 6 animals/group are provided as mean ± SEM. A two-way ANOVA was used to assess the effects of DHT and litter size and their interaction, followed by the Tukey *post hoc* test in a case of interaction between the factors. Statistical significance was accepted at *p* < 0.05. Symbols represent a significant difference in comparison with the NL-Placebo group or NL-DHT group (** *p* < 0.01 and ^##^ *p* < 0.01, respectively).

The gene expression of proinflammatory cytokine IL1β was affected by litter size (F(1, 18) = 16.67, *p* < 0.001) and its interaction with DHT (F(1, 18) = 4.60, *p* < 0.05), while the Tukey *post hoc* test showed a significant increase in the gastrocnemius muscle of SL-DHT animals in comparison with NL-Placebo and NL-DHT group (** *p* < 0.01; ^##^ *p* < 0.01; respectively) (Figure 7a).

The expression of the gene encoding another proinflammatory cytokine, IL6, was affected by litter size (F(1, 18) = 12.01, *p* < 0.01) and DHT treatment (F(1, 18) = 6.46, *p* < 0.05), as shown in Figure 7b. The combination of these two factors showed a trend of statistical significance (F(1,18) = 3.01, *p* = 0.10), indicating a tendency towards the elevation of this cytokine in the gastrocnemius muscle of SL-DHT rats.

## 4. Discussion

PCOS is primarily recognized and treated as a reproductive disorder, but increasing attention is being paid to its metabolic implications since a large proportion of women with PCOS are obese and have insulin resistance. Previous studies indicate a close interaction between androgen excess and insulin resistance, but the exact mechanism and direction of causality between these conditions are still unclear [2,25].

As previously mentioned, a more recent hypothesis emerged supporting the concept that there may be “PCOS secondary to obesity” and that prepubertal obesity might be a preconditioned state for hyperandrogenemia [18]. Namely, prepubertal obesity is believed to be a risk factor for the development of adolescent PCOS via insulin resistance and compensatory hyperinsulinemia, which increases ovarian/adrenal androgen production and suppresses sex hormone–binding globulin, thereby increasing androgen bioavailability [8,30]. This concept is corroborated by the fact that increasing childhood obesity is associated with an alarming prevalence of PCOS in adolescent girls, as well as severe impairment of glucose tolerance and the occurrence of T2DM at this age [3]. Being particularly interested in the association between prepubertal obesity and hyperandrogenemia in PCOS-associated metabolic disturbances, we introduced early postnatal overfeeding to a well-established DHT-induced rodent model of PCOS [28,31,32]. Recent animal studies have shown that early postnatal feeding of pups raised in a small litter is associated with increased caloric intake and that this model resembles children and adolescents with excessive caloric intake [9,13]. It is clinically recognized that such children are susceptible to developing metabolic syndrome and T2DM, which begins in adolescence and persists throughout life [10]. As mentioned earlier, prepubertal obesity is considered a risk factor for the development of PCOS, and even though it is neither sufficient nor necessary for its development, it is an important factor contributing to the presence and severity of the syndrome [8,30]. Thus, puberty represents a critical developmental window during which obesity-induced hyperinsulinemia and hyperandrogenemia may promote PCOS development [33]. Postnatal overfeeding, as a model for prepubertal obesity in rodents, is achieved by reducing litter size on the second *postpartum* day. It was shown that reduced competition between animals raised in small litters may increase the consumption of milk, which leads to a higher caloric intake compared to animals reared in normal litters [13,34]. Moreover, early postnatal overfeeding leads to misprogramming of the HPA axis and a lifelong increase in food consumption, which together should result in overweight or obesity [13]. Indeed, in our previous study, we showed that overfeeding induced by litter size reduction resulted in higher caloric intake, increased body mass, and visceral adiposity, but not obesity [35]. Our results showed that only when DHT treatment and overfeeding were combined, decreased systemic insulin sensitivity and hyperinsulinemia were present, which is likely a compensatory mechanism for maintaining normal glucose levels [35]. While the combination of both factors is crucial for promoting hyperinsulinemia, the effect of postnatal overfeeding on the increased glucose excursion during ipGTT is more dominant, suggesting the predisposing role of prepubertal adiposity in the development of PCOS-associated insulin resistance [25,36].

Given the central metabolic role of skeletal muscle in insulin-mediated glucose disposal, disruption of insulin signaling in this tissue is considered one of the possible major causes of systemic insulin resistance in PCOS [24]. Although the reduction in insulin-stimulated glucose uptake in women with hyperandrogenism is primarily due to skeletal muscle insulin resistance, other tissues, such as the endometrium, also contribute to systemic insulin resistance [4]. There are studies suggesting the presence of tissue-specific insulin resistance in PCOS, in which metabolic tissues are resistant to insulin while the ovaries remain sensitive. The compensatory hyperinsulinemia in the state of insulin resistance acts as a co-gonadotropin and stimulates androgen biosynthesis in the ovary, further contributing to hyperandrogenemia and forming a vicious cycle [6].

Since IRS and AKT are recognized as critical nodes of the insulin signaling pathway [37], we examined the expression and phosphorylation status of these proteins in the gastrocnemius muscle. We observed that postnatal overfeeding resulted in increased phosphorylation of IRS1 at Serine 307 and decreased relative expression of pAKT-Ser473, while pAKT-Thr308 expression, total AKT levels, and their ratio remained unchanged. These results suggest a causal role of postnatal overfeeding *per se* in the development of insulin resistance in skeletal muscle because both inhibitory phosphorylation of IRS1 at Serine 307 and activating phosphorylation of AKT at Serine 473 were affected. In line with these results, Shelley et al. [38] showed that female mice born to obese dams had reduced total IRS1 and AKT phosphorylation at Serine 473 in skeletal muscle. However, the reduced glucose transport, as assessed by decreased protein level of GLUT4, was observed only when DHT was present, which implies that the existence of both factors leads to early manifestation of muscle insulin resistance. The reduced insulin-mediated glucose disposal as a result of hyperandrogenemia was previously reported in both lean and obese insulin-resistant women with PCOS [23]. The observed impairment of insulin signaling in the muscle is most likely an early triggering factor for decreased systemic insulin sensitivity observed in postnatally overfed DHT-treated animals.

In the condition of insulin resistance and reduced glucose disposal, skeletal muscles switch to lipids as a fuel source [39]. We have previously demonstrated increased lipolysis in the subcutaneous adipose tissue of postnatally overfed DHT-treated animals, however, this was not followed by increased free fatty acids (FFAs) levels in the circulation [35]. Taking into account the results from the present study, it seems that the released FFAs were most likely transported to the muscle to serve as an alternative source of energy, which is corroborated by an increased level of FATP1 transporter observed in the skeletal muscle of these animals. In the muscle, FATP1 may interact with CPT1 contributing to fatty acid import into mitochondria, which leads to increased fatty acid oxidation [40]. This is in accordance with our results since both FATP1 gene expression and CPT1 protein level were increased after combined treatment, implying the prevalence of mitochondrial β-oxidation over lipogenesis and accumulation of lipids. The accumulation of lipids was also disfavored through reducing effects of DHT on the perilipin 2 protein level, which is positively correlated with the intracellular lipid content, and through decreased gene expression of Scd1, which leads to the accumulation of ceramides [41]. SCD1 global knockout mice were shown to exhibit increased CPT1 activity and β-oxidation [42] while perilipin 2^−/−^ myotubes showed a shift in metabolic energy utilization towards fatty acid oxidation [43]. In addition, DHT treatment was shown to be associated with decreased lipid accumulation and cholesterol synthesis in the liver through increased expression of CPT1 via an androgen receptor-mediated pathway [44]. Changes in lipid metabolism, observed in the postnatally overfed DHT-treated animals, were also accompanied by upregulated lipolytic enzyme ATGL, recognized as an important factor for preventing lipid accumulation [45]. Altogether, these results suggest that the combination of treatments used in this study prevents the accumulation of intramyocellular lipids and instead leads towards β-oxidation of fatty acids.

The presence of muscle oxidative stress is often associated with mitochondrial dysfunction and redox imbalance, as demonstrated in the skeletal muscles of mice with PCOS [46]. This could lead to the activation of AMPK as part of the adaptive response to increased oxidative stress and has the function of attenuating oxidative injury [47]. Activation of AMPK by oxidative stress leads to metabolic reprogramming of skeletal muscle and regulates the expression of genes involved in mitochondrial biogenesis, antioxidant defense, and glycolysis [48]. In this study, when DHT treatment and postnatal overfeeding were combined, increased oxidative stress and, in parallel, induced AMPK activation, as evidenced by its increased phosphorylation at Threonine 172, were observed. While the stimulation of AMPK activity in this study was dependent on the existence of both factors, expression of the master regulators of AMPK-driven mitochondrial biogenesis [49], PGC1α and SIRT1, was dominantly regulated by postnatal overfeeding, which implies some AMPK-independent regulation of mitochondrial function in the postnatally overfed animals [50]. The additional factors that contribute to the AMPK activation after combined treatment are increased cytokines in these animals, since *Il1b*, as obesity-related, and *Il6*, as a muscle energy sensor, contribute to increased AMPK activity [51,52]. The increased activity of AMPK and its downstream targets, SIRT1 and PGC1α, has been shown to increase the expression of SOD2 and catalase in skeletal muscle, thereby enhancing antioxidant defense [53]. Our results showed that postnatal overfeeding was associated with increased expression of SOD2, which was most prominent in the postnatally fed DHT-treated animals, where the MDA level was significantly increased. Overall, stimulated AMPK activity along with increased expression of SIRT1 and PGC1α, as well as an increased level of the antioxidant enzyme SOD2, were observed only after combined treatment, implying metabolic reprogramming of muscle cells in coping with oxidative stress and inflammation.

In summary, our results clearly demonstrate a prooxidative and proinflammatory milieu in the skeletal muscle of rats exposed to early postnatal overfeeding and DHT treatment, which may be the triggers for the insulin resistance observed at this tissue level. Insulin resistance in skeletal muscle may occur much earlier than systemic hyperglycemia [26], and given the central metabolic role of skeletal muscle, it is considered a critical organ for the progression of systemic insulin resistance in PCOS [2,25]. Our results indicate that postnatal overfeeding is a contributing factor to impaired insulin signaling in skeletal muscle, whereas the presence of both overfeeding and hyperandrogenemia leads to more profound insulin resistance. The prooxidative and proinflammatory state in the muscle of postnatally overfed DHT-treated animals is most likely the result of excessive β-oxidation and is accompanied by the activation of AMPK and increased expression of regulators of mitochondrial biogenesis and antioxidant defense. The stimulation of these processes by activated AMPK probably has beneficial effects on skeletal muscle function under insulin-resistant conditions. Since skeletal muscle dysfunction contributes most to the impairment of systemic insulin sensitivity and the compensatory occurrence of hyperinsulinemia, future research should address in depth the pathophysiological processes in the skeletal muscle of obese individuals with PCOS and identify potential therapeutic targets. Overcoming insulin resistance in PCOS at the level of key insulin target tissues such as skeletal muscle would suppress compensatory hyperinsulinemia and thus indirectly reduce insulin-stimulated androgens overproduction and their bioavailability, thereby improving androgen levels in PCOS. Since endometrial receptivity may be reduced in PCOS due to insulin resistance [4], future research to discover ways to activate AMPK in the endometrium could improve pregnancy prospects in PCOS women. According to our results and available data, AMPK undoubtedly represents an important target for therapeutic intervention in insulin-resistant PCOS patients, both in terms of medication and lifestyle intervention. We also note that early postnatal overfeeding, introduced in this study as an adjunct to the well-established DHT-induced PCOS model, is a valuable tool for a better understanding of the interplay between obesity and hyperandrogenemia in the development of the deleterious metabolic phenotype of PCOS.

## Data Availability

The data presented in this study are available on request from the corresponding authors.
